# Future Prospects for *Neisseria gonorrhoeae* Treatment

**DOI:** 10.3390/antibiotics7020049

**Published:** 2018-06-15

**Authors:** Beatriz Suay-García, María Teresa Pérez-Gracia

**Affiliations:** Área de Microbiología, Departamento de Farmacia, Instituto de Ciencias Biomédicas, Facultad de Ciencias de la Salud, Universidad CEU Cardenal Herrera, C/Santiago Ramón y Cajal, 46115 Alfara del Patriarca, Valencia, Spain; bea.suay.ce@ceindo.ceu.es

**Keywords:** *Neisseria gonorrhoeae*, antibiotic resistance, gonorrhea, treatment

## Abstract

Gonorrhea is a sexually transmitted disease with a high morbidity burden. Incidence of this disease is rising due to the increasing number of antibiotic-resistant strains. *Neisseria gonorrhoeae* has shown an extraordinary ability to develop resistance to all antimicrobials introduced for its treatment. In fact, it was recently classified as a “Priority 2” microorganism in the World Health Organization (WHO) Global Priority List of Antibiotic-Resistant Bacteria to Guide Research, Discovery and Development of New Antibiotics. Seeing as there is no gonococcal vaccine, control of the disease relies entirely on prevention, diagnosis, and, especially, antibiotic treatment. Different health organizations worldwide have established treatment guidelines against gonorrhea, mostly consisting of dual therapy with a single oral or intramuscular dose. However, gonococci continue to develop resistances to all antibiotics introduced for treatment. In fact, the first strain of super-resistant *N.* *gonorrhoeae* was recently detected in the United Kingdom, which was resistant to ceftriaxone and azithromycin. The increase in the detection of resistant gonococci may lead to a situation where gonorrhea becomes untreatable. Seeing as drug resistance appears to be unstoppable, new treatment options are necessary in order to control the disease. Three approaches are currently being followed for the development of new therapies against drug-resistant gonococci: (1) novel combinations of already existing antibiotics; (2) development of new antibiotics; and (3) development of alternative therapies which might slow down the appearance of resistances. *N. gonorrhoeae* is a public health threat due to the increasing number of antibiotic-resistant strains. Current treatment guidelines are already being challenged by this superbug. This has led the scientific community to develop new antibiotics and alternative therapies in order to control this disease.

## 1. Introduction

**Figure 1:**
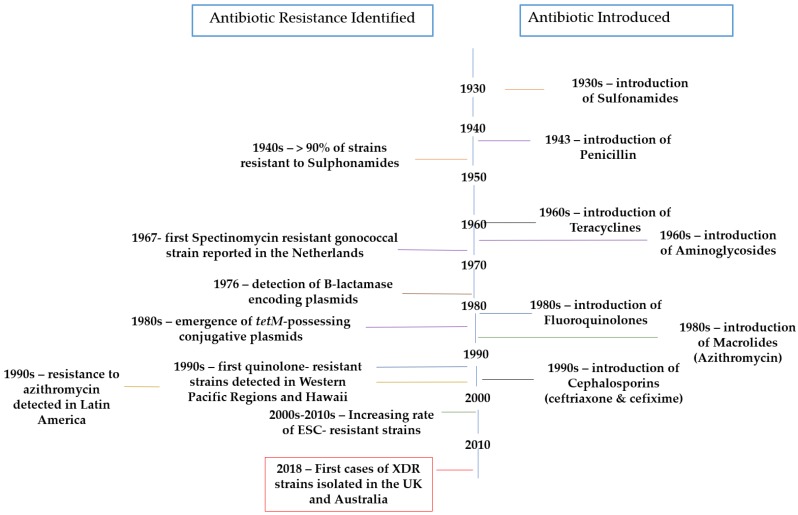
Timeline representing the introduction of treatments used against gonorrhea (right) and the first reports of resistance (left) [4,7,8].

Gonorrhea is a sexually transmitted disease (STD) caused by the obligate human pathogen *Neisseria gonorrhoeae*. This disease has a high morbidity burden, with more than 106 million new cases being diagnosed every year worldwide [1]. In fact, this morbidity is increasing exponentially due to the fact that gonococci have an extraordinary ability to develop resistances to all antimicrobials introduced for its treatment (Figure 1) [2,3,4].

The issue with drug-resistant *N. gonorrhoeae* has become such that the Centers for Disease Control (CDC) classified it as a “superbug” in 2012, announcing a near future in which gonorrhea would become untreatable [5]. Furthermore, the World Health Organization (WHO) classified it as a “Priority 2” microorganism in the recently published WHO Global Priority List of Antibiotic-Resistant Bacteria to Guide Research, Discovery, and Development of New antibiotics [6]. This document highlights the importance of developing new antibiotics to treat this disease, seeing as the existence of *N. gonorrhoeae* strains resistant to third generation cephalosporins and fluoroquinolones have already been reported. In fact, failure of current dual therapy was detected in the United Kingdom in 2016. More importantly, the first “super-resistant” strains were recently reported in the United Kingdom and Australia, showing resistance against the current first line treatment, dual therapy with azithromycin and ceftriaxone [7,8].

Seeing as there is no gonococcal vaccine, control of the disease relies entirely on prevention, diagnosis, and, especially, antibiotic treatment [9]. It is for this reason that the present review focuses on current treatment options and the future perspectives for the treatment of this disease.

## 2. Current Treatment

Generally, treatment for gonococcal infection is given at the first clinical visit, which implies that antimicrobial susceptibility is rarely performed prior to prescription. According to WHO guidelines [10], first-line antimicrobial therapy must be highly effective, widely available and affordable, lack toxicity, comprise a single dose, and rapidly cure at least >95% of infected patients.

Different health organizations worldwide have established treatment guidelines against gonorrhea, mostly consisting of dual therapy with a single oral or intramuscular dose of a third-generation cephalosporin (250–500 mg intramuscular (IM) ceftriaxone or 400 mg per os-oral (PO) cefixime) in combination with a single oral dose of 1–2 g of azithromycin [11,12,13,14,15,16,17] (Table 1).

However, as it was mentioned earlier, these treatment options will not be useful in the near future, as they have already been reported as ineffective in treating some patients [7,8,18]. With this in mind, it is evident that, in the absence of a vaccine, the future control of this disease relies completely on the development of new antibiotics and alternative treatments.

## 3. Future Perspectives

Seeing as drug resistance appears to be unstoppable, new treatment options are necessary in order to control the disease [19]. Three approaches are currently being followed for the development of new therapies against drug-resistant gonococci: (1) novel combinations of already existing antibiotics; (2) development of new antibiotics; and (3) development of alternative therapies, which might slow down the appearance of resistances (Table 2).

### 3.1. Repurposing of Already Existing Antibiotics

Considering the fact that untreatable gonorrhea has indeed become a reality, the need for new treatment options has become a pressing issue. For this reason, the scientific community has turned to trying new combinations of already existing antibiotics as the fastest way to fight multi-resistant superbugs. Along these lines, Jönsson et al. studied the viability of introducing sitafloxacin, a newer-generation broad spectrum fluoroquinolone mostly used for respiratory infections, as part of a dual therapy against gonococci [20]. In the study, sitafloxacin was tested against a global gonococcal panel of 250 isolates, showing a rapid bactericidal effect with a Minimum Inhibitory Concentration (MIC) range of ≤0.001–1 mg/L. These results prove that sitafloxacin is a good candidate to be included in dual antimicrobial therapy for gonorrhea in cases with cephalosporin resistance or allergy.

Along these lines, another study focuses on the evaluation of sitafloxacin and five additional fluoroquinolones against ciprofloxacin-resistant *N. gonorrhoeae* isolates [21]. The in vitro potency of sitafloxacin was substantially higher compared with the other five fluoroquinolones, with an MIC range of 0.03–0.5 mg/L against the ciprofloxacin-resistant strains. These results further confirm the utility of sitafloxacin in dual antimicrobial therapy.

Another fluoroquinolone currently being studied for the treatment of gonorrhea is delafloxacin [22]. Soge et al. evaluated the activity of delafloxacin against 117 strains of *N. gonorrhoeae*. The results showed an MIC range of ≤0.001–0.25 µg/mL, which is higher than that of ciprofloxacin, penicillin, tetracycline, azithromycin, and spectinomycin. Further studies are required to correlate these promising in vitro results with clinical treatment outcomes.

On a similar note, Singh et al. assayed the potent utility of in vitro interactions of 21 dual therapy combinations against 95 *N. gonorrhoeae* strains [23]. Of these 21 combinations, five were novel introductions that are not included in any existing guidelines: gentamicin + ertapenem, moxifloxacin + ertapenem, spectinomycin + ertapenem, azithromycin + moxifloxacin, and cefixime + gentamicin. All five novel combinations produced high synergistic effects against the studied strains, which suggests that further in vivo evaluation in clinical trials should be performed in order to include these combinations for future treatment of gonorrhea.

Gentamicin is already included in several guidelines in combination with azithromycin as an alternative treatment option when main treatment options fail. A recent study examines the synergistic effect of this combination along with gentamicin combined with five other antimicrobials (cefixime, ceftriaxone, spectinomycin, azithromycin, moxifloxacin, and ertapenem) [24]. The study concludes that gentamicin in combination with ertapenem or cefixime could be introduced as new antimicrobial dual therapy seeing as these combinations showed maximum efficacy and synergism against 75 gonococcal strains.

### 3.2. New Antibiotics

However, seeing as gonococci have proven to be able to develop resistances to all antibiotics introduced for their treatment, the long-term solution includes the development of new antibiotics. Ideally, these new antibiotics should belong to antibacterial families different to the ones already included in treatment guidelines in order to delay the appearance of resistances as much as possible.

Along these lines, WHO launched the Global Antibiotic Research and Development Partnership (GARDP) in order to work with experts to draw a plan to meet the urgent need for new drugs to treat gonorrhea [25]. Within this partnership, experts analyze current drugs in clinical development for the treatment of this disease. Currently, only three molecules have reached clinical trials: Solithromycin, Zoliflodacin, and Gepotidacin.

Solithromycin is a broad-spectrum oral fluoroketolide which targets three prokaryotic ribosomal sites [26]. In vitro studies against 246 clinical isolates and international reference strains of *N.* gonorrhoeae showed promising results, with an MIC range of 0.001–32 µg/mL, showing more activity than the antimicrobials currently recommended for its treatment. Phase II clinical trials concluded with 100% efficacy for infection in men and women for all studied sites (genital, oral, and rectal) [27]. This drug is currently in Phase III trials.

As for Zoliflodacin, it has a novel action mechanism by which it inhibits the spiropyrimidinetrione topoisomerase [28]. Early in vitro studies showed promising results, with the compound being highly effective against clinical isolates from 21 European countries [29]. Zoliflodacin showed an MIC range of ≤0.002–0.25 µg/mL, considerably lower than that of most drugs currently being used for treatment but, most importantly, it did not present any cross-resistance to these antimicrobials.

Similarly, Farrell et al. studied the antigonococcal activity of Gepotidacin, a novel triazaacenaphthylene antibacterial which inhibits bacterial DNA gyrase and topoisomerase IV via a unique mechanism [30,42]. The compound had an MIC_50_ and MIC_90_ of 0.12 and 0.25 mg/L, respectively, against 25 *N. gonorrhoeae* strains, including five ciprofloxacin non-susceptible strains. Moreover, synergism studies showed that no antagonism occurred when gepotidacin was combined with levofloxacin, azithromycin, tetracycline, and ceftriaxone; while the combination of gepotidacin with moxifloxacin had a synergistic effect. This drug candidate underwent a Phase II evaluation, showing that oral doses of gepotidacin were ≥95% effective in treating uncomplicated urogenital gonorrhea [31].

Along with these three drugs in clinical trials, other compounds being developed to treat gonorrhea are still in early experimental phases. This is the case of Lefamulin, a novel semi-synthetic pleuromutilin, recently evaluated against 251 gonococcal clinical isolates, including multidrug-resistant and extensively-drug resistant samples [32]. The compound showed potent activity, an MIC range of 0.004–2 mg/L, against gonococcal isolates and no significant cross-resistance to other antimicrobials. Furthermore, this compound has also been proven to be active against the other most relevant bacterial pathogens causing sexually transmitted infections (STIs), *Chlamydia trachomatis* and *Mycoplasma genitalium*, proving to be a good candidate first-line antibiotic for the treatment of STIs [33]. However promising, further studies are required in order to consider the introduction of Lefamulin as a first-line treatment option.

For that matter, Butler et al. studied aminoethyl spectinomycins, a new class of semisynthetic analogs of the antibiotic spectinomycin, for the treatment of drug-resistant gonococci [34]. The studied compounds presented increased potency against *N. gonorrhoeae* compared to spectinomycin. Furthermore, these compounds also demonstrated activity against *C. trachomatis*, which is not observed with spectinomycin. The study concludes that aminoethyl spectinomycins are a promising alternative for spectinomycin and antibiotics such as ceftriaxone against drug-resistant gonorrhea, with the added benefit of treating chlamydial co-infections.

Furthermore, novel antibacterials in the earliest stages of drug design and screening have also been reported. Fedarovich et al. screened a 50,000 compound library for potential inhibitors of *N. gonorrhoeae* penicillin binding protein 2 (PBP 2) using fluorescence polarization [35]. The screening resulted in 32 compounds exhibiting >50% inhibition of Bocillin-FL binding to PBP 2, of which seven showed antimicrobial activity against susceptible and penicillin- or cephalosporin-resistant strains. These seven molecules remain as lead compounds for future optimization as anti-gonococcal agents.

### 3.3. Alternative Therapies

In addition to new antibiotics, alternative therapies to combat increasingly resistant *N. gonorrhoeae* are being developed. These alternatives are mainly focused on the prevention of recurring infections rather than on the treatment of the disease. In this regard, early in vivo studies have been performed regarding the intravaginal administration of interleukin-12 (IL-12) in mice [36]. The study concludes that intravaginally administered IL-12 promotes the Th1-driven adaptive immune response, including the production of specific anti-gonococcal antibodies which would prevent recurring infection.

On a similar note, Foschi et al. studied the efficacy of vaginal lactobacilli in reducing *N. gonorrhoeae* viability [37]. The study assessed the anti-gonococcal activity of 14 vaginal *Lactobacillus* strains belonging to *L. crispatus, L. gasseri*, and *L. vaginalis*. It was found that the acidic environment associated with lactobacilli metabolism is extremely effective in counteracting gonococcal growth, with complete abolishment of gonococci viability being observed at pH < 4.0. Furthermore, results showed that lactobacilli cells are able to reduce viability and co-aggregate with gonococci. This is achieved by released-surface components with biosurfactant properties produced by lactobacilli. The study concludes that specific *Lactobacillus* strains, mainly belonging to *L. crispatus*, are able to counteract gonococcal viability through multiple mechanisms, representing a new potential probiotic strategy for the prevention of infection in women.

Prophylaxis is especially important during pregnancy, seeing as neonatal conjunctivitis is commonly caused by *N. gonorrhoeae* [38]. The most common approach is ophthalmic prophylaxis with antibiotic ointments. However, due to the increasing appearance of resistances, these are becoming less effective. Churchward et al. studied 37 fatty acids or fatty acid derivatives for fast antigonococcal activity [39]. Two lead candidates, monocaprin and myristoleic acid, were bactericidal at 1 mM and remained active in artificial tear fluid, becoming promising alternatives to conventional antibacterial ointments. They went on to study the ability of *N. gonorrhoeae* to develop resistance when grown in sub-lethal concentrations of monocaprin [43]. Results showed that, after growing gonococci on growth media containing sub-lethal concentrations of monocaprin, the MIC showed a two-fold change, which cannot be considered as the development of resistance. Thus, the study concludes that *N. gonorrhoeae* in not capable of developing resistances against monocaprin, making it an ideal long-term alternative for neonatal conjunctivitis prophylaxis.

Another alternative treatment that has gained importance recently is bacteriophage therapy, as a therapeutic option on its own and also in combination with currently used antimicrobials [44,45]. However promising, this type of therapy is still in early stages when it comes to treating gonorrhea. Experiments with peptide inhibitors targeting gonococci identified using phage display have been reported in recent years [40,41]. Connor et al. constructed open reading frame phagemid (pHORF) oligopeptide phage display libraries of the entire *N. gonorrhoeae* genome, identifying six immunogenic proteins for the first time and verifying 13 additional proteins as immunogenic in *N. gonorrhoeae* [40]. Similarly, Sikora et al. focused on targeting the nitrite reductase AniA, a key component of gonococcal anaerobic respiration and biofilm formation [41]. One of the 29 unique peptides identified, C7-3, and its derivative (C7-3m2), demonstrated potent inhibition of AniA, with an MIC50 value of 0.6 mM against anaerobically grown *N. gonorrhoeae*. These studies show promising results towards the development of bacteriophage therapy for the treatment of gonorrhea; however, further studies are required in this field.

## 4. Conclusions

*Neisseria gonorrhoeae* is a public health threat worldwide due to the increasing number of antibiotic-resistant strains. Current treatment guidelines include first-line treatments, as well as alternative treatments which should only be prescribed in case of allergy or presence of resistance. However, most of these guidelines are already being challenged by this “Superbug”. This has led the scientific community to develop new antibiotics and alternative therapies in order to control this disease. These new treatment options require not only high antigonococcal potency, but also no cross-resistance with current antibiotics in order to assure their applicability in the long-run. Alternative therapies, on the other hand, have focused on preventing infections rather than treating them and, therefore, controlling the disease before it has a chance of developing further resistances.

## Figures and Tables

**Table 1:** Different treatment guidelines for gonorrhea worldwide (all single dose).

WHO * [11]	Australasia [12]	Canada [13]	USA [14]	UK [15]	EU [16]	New Zealand [17]
Ceftriaxone 250 mg IM + Azithromycin 1 g PO Or ** Cefixime 400 mg PO + Azithromycin 1 g PO	Cetriaxone 500 mg IM + Azithromycin 1 g PO	Ceftriaxone 250 mg IM + Azithromycin 1 g PO	Ceftriaxone 250 mg IM + Azithromycin 1 g PO	Ceftriaxone 500 mg IM + Azithromycin 1 g PO	Ceftriaxone 500 mg IM + Azithromycin 1 g PO	Ceftriaxone 250 mg IM + Azithromycin 1 g PO
Ceftriaxone 500 mg IM + Azithromycin 2 g PO Or ** Cefixime 800 mg PO + Azithromycin 2 g PO Or ** Gentamicin 240 mg IM + Azithromycin 2 g PO Or ** Spectinomycin 2 g IM + Azithromycin 2 g PO		Cefixime 800 mg PO + Azithromycin 1 g PO Or ** Spectinomycin 2 g IM + Azithromycin 1 g PO	Cefixime 400 mg PO + Azithromycin 1 g PO	Cefixime 400 mg PO + Azithromycin 1 g PO Or ** Spectinomycin 2 g IM + Azithromycin 1 g PO Or ** Cefotaxime 500 mg IM + Azithromycin 1 g PO	Cefixime 400 mg PO + Azithromycin 2 g PO Or ** Spectinomycin 2 g IM + Azithromycin 2 g PO	Spectinomycin 2 g IM + Azithromycin 1 g PO Or ** Gentamicin 240 mg IM + Azithromycin 2 g PO

* WHO (World Health Organization); IM (Intramuscular); PO (Per os-oral) ** An “or” between combinations means that any of those combinations may be prescribed.

**Table 2:** Antigonococcal agents currently under development.

Future Options	Name	Action Mechanism	Structure	Reference
Drug repurposing	Sitafloxacin	DNA gyrase and topoisomerase IV inhibitor	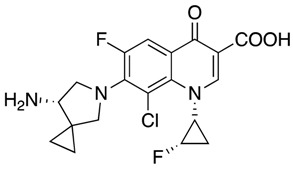	[20,21]
	Delafloxacin	DNA gyrase and topoisomerase IV inhibitor	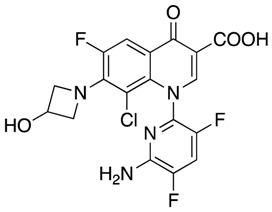	[22]
	Novel dual therapies	-	-	[23,24]
New antibacterial agents	Solithromycin	Protein synthesis inhibitor	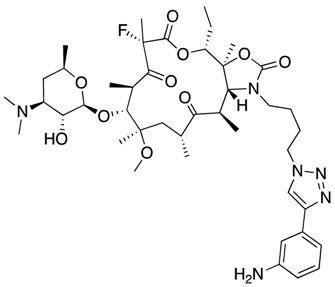	[25,26,27]
	Zoliflodacin	Spiropyrimidinetrione topoisomerase inhibitor	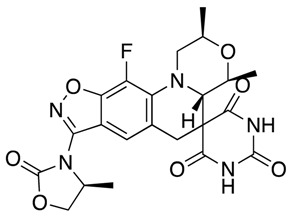	[28,29]
	Gepotidacin	DNA gyrase and topoisomerase IV inhibitor	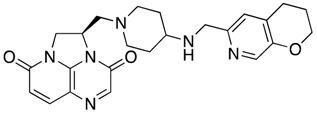	[25,30,31]
	Lefamulin	Protein synthesis inhibitor	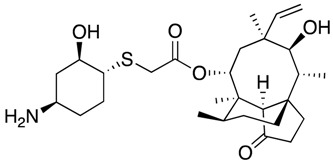	[32,33]
	Aminoethyl spectinomycins	Protein synthesis inhibitor	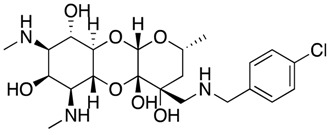	[34]
	PBP2 inhibitors	Inhibition of cell wall synthesis	-	[35]
Alternative therapies	IL-12	Induction of immune response	-	[36]
	*Lactobacillus crispatus*	Biosurfactant and acidic environment	-	[37]
	Monocaprin Myristoleic acid	Cell membrane disruption	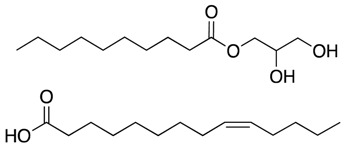	[38,39]
	Bacteriophage therapy	Lysis	-	[40,41]
